# Prevalence and Determinants of Anemia Among Women of Reproductive Age and Children Under Five in Tajikistan

**DOI:** 10.3390/nu18050752

**Published:** 2026-02-26

**Authors:** Sajid B. Soofi, Imran A. Chauhadry, Imtiaz Hussain, Muhammad Atif Habib, Muhammad Umer, Shabina Ariff, Lailo Kurbonmamadova, Nizoramo Ramikhudoeva, Roziya Buribekova, Aminah Jahangir, Claudia Hudspeth, Zulfiqar A. Bhutta

**Affiliations:** 1 Centre of Excellence in Women and Child Health, Aga Khan University, Karachi 74800, Pakistan; 2 Department of Pediatrics & Child Health, Aga Khan University, Karachi 74800, Pakistan; 3 Aga Khan Foundation, Dushanbe 734003, Tajikistan; 4 Aga Khan Health Services, Dushanbe 734025, Tajikistan; 5 Aga Khan Foundation, 1202 Geneva, Switzerlandclaudia.hudspeth@akdn.org (C.H.); 6 Institute of Global Health & Development, Aga Khan University, Karachi 74800, Pakistan; 7 Departments of Paediatrics, Nutritional Sciences & Public Health, University of Toronto, Toronto, ON M5S 1A1, Canada

**Keywords:** anemia, iron deficiency, children under five, women of reproductive age, preschool-age children, GBAO, Tajikistan

## Abstract

**Introduction:** Anemia is a global public health concern affecting mainly women of reproductive age (WRA) and preschoolers (PSC, 6–59 months) due to their higher demand for iron. The etiology of anemia is multifactorial, and nutritional anemia is the most common type worldwide and is predominantly due to deficiencies in iron, vitamin B12, and folate. This study aimed to assess the associated factors, etiology, and prevalence of anemia among WRA and preschoolers in the Gorno-Badakhshan Autonomous Oblast (GBAO) region in Tajikistan. **Methods:** We conducted a cross-sectional study in the GBAO region of Tajikistan between April 2021 and September 2021, enrolling 500 non-pregnant WRA and 500 children aged 6–59 months in pairs (mother–PSC dyads) from six districts of the GBAO region. The survey was administered through a structured questionnaire at the household level, and information was collected on sociodemographic characteristics, reproductive history, dietary intake, nutritional status, and maternal factors. Blood and stool samples were also collected for micronutrient deficiencies and helminthic infections. Data analysis began with univariate analysis, followed by multivariate logistic regression in Stata (version 18). **Results:** Biochemical assessment of 473 WRA and 390 preschoolers was carried out; 17.3% of WRA were anemic, and 15.4% of PSC were anemic. Anemia prevalence was 17.3% among WRA and 15.4% among PSC. Among women, low ferritin and elevated serum transferrin receptor (sTfR) levels were associated with higher odds of anemia, whereas overweight status, higher gravidity, and vitamin B12 deficiency were associated with lower odds. Among children, low maternal education, maternal anemia, age < 24 months, and low ferritin were associated with increased odds of anemia. **Conclusions**: Anemia prevalence in GBAO was substantially lower than reported in the 2016 national survey, potentially reflecting methodological and contextual differences. Findings highlight iron deficiency as a dominant contributor, particularly in young children, underscoring the need for context-specific maternal and child nutrition interventions.

## 1. Background

Anemia is characterized by low levels of hemoglobin (HB), in which the number of red blood cells are inadequate to satisfy the physiological requirements of the body [1]. This condition has become a global public health concern; it can be identified when the HB levels in the blood are decreased to a predetermined threshold of (<12.0 g/dL) in non-pregnant women of reproductive age (15–49 years) and (<11.0 g/dL) in children aged 6–59 months, thereby impairing the ability of the blood to transport oxygen to the body [2]. This crucial matter has gained global attention owing to its impact on various groups of individuals, mainly women in their reproductive years (aged 15–49) and young children (aged 6–59 months). Anemia continues to threaten women during pregnancy and can be associated with a heightened risk of maternal mortality that can lead to preterm delivery, low birth weight, and cognitive development difficulties [3]. The etiology of anemia is complex; its prevalence and reasons extend substantially worldwide based on population groups, geographical location, socioeconomic status (SES), and other factors [4]. The population primarily residing in rural households with low socioeconomic status and having inadequate necessities of life, including proper sanitation and safe water provision, is more susceptible to anemia [5]. Furthermore, micronutrient deficiencies, particularly iron, folate, and vitamin B12 deficiencies, can also be common causes of anemia among women in reproductive years and preschool-age children (PSC—6–59 months) [6,7].

The World Health Assembly has set a goal to reduce the burden of anemia among WRA to 50% by 2025 [8]. According to the 2019 findings of the World Health Organization, at a global scale, anemia was prevalent in 29.9% of women of reproductive age and in 39.8% of children aged 6–59 months [5,9]. Anemia accounts for maternal and neonatal deaths of 2.5 million to 3.4 million deaths worldwide [10]. Additionally, many estimates indicate a significant prevalence of anemia in women and young children, especially in low- and middle-income countries (LMICS) [11]. Anemia affects developing countries in disproportionate numbers, with a burden of 89% in Asian and sub-Saharan African countries [3]. In addition, Kazakhstan, a landlocked country in Central Asia, reported anemia prevalence of 29% in WRA and 23% in preschool-age children (PSC—6–59 months) in 2019 [12]. Furthermore, Uzbekistan, another country in Central Asia, reported an anemia prevalence of 25% in WRA, while 22% of preschool children (6–59 months) had anemia [13]. Like other low- to middle-income countries, Tajikistan, geographically located in Central Asia, bears the burden of anemia. According to the Tajikistan National Nutrition Survey 2016, anemia affected 25.8% of children aged 6–59 months and 20.7% of women of reproductive age (15–49 years) at the national level. In GBAO, the prevalence was substantially higher, affecting 43.4% of children and 30.0% of women [14]. The underlying causes of anemia among WRA and children are evaluated as multifactorial, as there is limited available data about factors associated with anemia in Tajikistan; therefore, this study has performed a survey on WRA, childhood anemia, and its determinants in which the prevalence of anemia, associated factors of anemia, and its etiology are discussed.

## 2. Methods

### 2.1. Survey Design

The cross-sectional survey was conducted at the household level in GBAO, Tajikistan. The data was collected from mothers (WRA-15–49 years, *n* = 500) and their preschool-age children (6–59 months, *n* = 500) in pairs (mother–PSC dyads), over the period of six months between April 2021 and September 2021. This survey was conducted in 6 districts of the GBAO region (Khorog, Murghab, Roshtkala, Rushan, Shugnan, Ishkisham). Apart from assessing anemia, other proposed indicators associated with anemia were also collected, followed by micronutrient deficiencies, socioeconomic status, demographic, dietary intake, and other maternal factors.

### 2.2. Sample Size and Sampling Design

The sample size of 500 women was calculated to estimate anemia prevalence with 5% precision, 95% confidence, and 80% power, accounting for a design effect of 1.5 and non-response.

A two-stage cluster sampling design was employed. In the first stage, six districts (Khorog, Murghab, Roshtkala, Rushan, Shugnan, and Ishkashim) were included. Four villages per district (total *n* = 24 villages) were randomly selected from updated administrative lists. In the second stage, a household listing was conducted in each selected village; 20 eligible households per village were randomly selected. Households were eligible if at least one child aged 6–59 months resided in the household. The biological mother of the eligible child was recruited. To satisfy the sample size of 500, we added one more village in the sample and it was randomly assigned to Shugnan.

### 2.3. Biochemical Assessment

Blood samples were collected from WRA (*n* = 473) and preschool-age children (PSC-6–59 months, *n* = 390) for the assessment of anemia. A standardized methodology was employed to collect a blood sample via venipuncture for biochemical evaluation of hemoglobin. A trained phlebotomist collected 3 mL of whole blood from the participant, centrifuged, and separated the serum at the field site, which was then transported to the lab for storage at 2–8 °C. The samples were then transported to the AKMC laboratory in Khorog under cold chain conditions. In addition, stool samples were examined using light microscopy for detection of helminth infections, specifically *Ascaris lumbricoides* and *Enterobius vermicularis (pinworm)*.

Hemoglobin was measured using an automated hematology analyzer. Serum ferritin, soluble transferrin receptor (sTfR), CRP, AGP, vitamin B12, and folate were measured using ELISA methods in accordance with standardized operating procedures with external quality assurance support from the Nutritional Research Lab at Aga Khan University, Pakistan. Stool samples were collected and examined in the field by trained personnel using light microscopy.

In non-pregnant WRA (15–49 years) and children (6–59 months), anemia was defined as hemoglobin levels < 12 g/dL and <11 g/dL, respectively. It is important to note that hemoglobin levels were altitude adjusted, along with ferritin levels and IDA, which were adjusted for CRP and AGP [15]. Furthermore, the survey data were collected using handheld devices via the computer-assisted Personal Interview (CAPI) technique; however, in areas where CAPI could not be used due to security concerns, data collection was conducted using the Paper-Based Interview (PAPI) approach. A structured household questionnaire was administered, including sociodemographic, nutrition, and reproductive information.

Anthropometric measurements were conducted using standardized procedures. Weight was measured using UNICEF-approved SECA digital scales, and height or length was measured using portable Shorr measuring boards. Children younger than 24 months were measured in the recumbent position, while standing height was measured for older children. Height- or length-based z-scores (HAZ/LAZ, WAZ, WHZ/WLZ) were calculated according to age. Stunting and wasting were defined as height-for-age (HAZ/LAZ) and weight-for-height/length (WHZ/WLZ) z-scores below −2 standard deviations of the WHO Child Growth Standards [15].

### 2.4. Ethical Consideration

Informed consent was obtained from study participants; however, written informed consent was obtained before blood samples were collected. Consent for the children was obtained from the mother. The study was approved by the Aga Khan University Ethics Review Committee (ERC# 2019-1582-4219 3 July 2019) and the Ministry of Health of the Republic of Tajikistan.

### 2.5. Measurement of Variables

#### 2.5.1. Outcome Variable

Anemia was defined as low hemoglobin levels.

#### 2.5.2. Explanatory Variable

According to literature and biological understanding, explanatory variables were incorporated into the analysis, including various socioeconomic, demographic, and health factors associated with WRA, as well as children’s nutritional status. Common associated factors included maternal age and education, household economic status (wealth quintile), food insecurity, dietary intake, micronutrient deficiencies, worm infestation, women’s BMI, and reproductive determinants.

### 2.6. Statistical Analysis

All data analyses were conducted in STATA version 18. Since anemic status was the primary outcome measure, women and children with available hemoglobin results were included in the study analysis. The frequencies, along with percentages, were reported for selected predictors. The analysis started with a simple univariate analysis followed by a multivariate logistic regression. Multivariable models were adjusted for variables with *p* < 0.25 in bivariate analysis and those considered biologically relevant, including maternal age, BMI, gravidity, ferritin, transferrin receptor, and vitamin B12. Unadjusted odds ratios with their 95% CIs were reported for the bivariate analysis. Variables significant at *p* < 0.25 were considered for inclusion in the multivariate model. The Type 1 error was set at 0.05. The model estimates are presented as adjusted odds ratios (AORs) with 95% CIs.

## 3. Results

### 3.1. Characteristics of Women of Reproductive Age (WRA, 15–49 Years)

A total of 500 women of reproductive age (15–49 years) were enrolled for this study in six districts of the GBAO region. Among all districts, Shugnan exhibited one-fifth of WRA in the region; conversely, other districts demonstrated an equal proportion. In particular, WRAs in the 30–34 age group were the highest (32.8%) among the sampled population (Table 1), whereas only 2.6% were identified in the 45–49 age group. Most of these women were married (97.8%), and 40.2% had higher education. In terms of occupation, the majority of WRA reported as a formal employee (39.4%). In contrast, nearly one-third of women were reported to be unemployed (28.2%), and one-fourth of WRA were reported to be housewives (24.8%).

When exploring housing characteristics, approximately 79% had access to improved drinking water sources. However, the majority of households (91.8%) had access to improved sanitation facilities. In addition, the food insecurity status indicated that 41.6% of households were food secure; conversely, 13.2% were moderately food insecure

Among women of reproductive age, 17.3% were anemic, while 19.4% had low ferritin levels, indicating a considerable burden of iron deficiency. Iron deficiency anemia was identified in 11.6% of participants. Folic acid deficiency was highly prevalent, affecting 61.6% of women, whereas vitamin B12 deficiency was observed in 11.2%. Elevated transferrin receptor levels were present in 22.0%, suggesting iron-deficient erythropoiesis, while elevated C-reactive protein, indicative of inflammation, was found in 6.6% of the study population. More than half of the participants (51.5%) tested positive for intestinal helminths on stool examination, with Ascaris detected in 38.7% and pinworm infection in 13.7%.

### 3.2. Factors Associated with Anemia (Women of Reproductive Age 15–49 Years)

Table 2 presents the bivariate and multivariable analyses of the association between various factors of anemia among WRA (15–49 years) in the GBAO region. Overall anemia prevalence was 17.3%. District-level differences were modest and not statistically significant; Ishkashim had the lowest prevalence (12%), while Roshtkala, Shugnan, Murghab, and Khorog town showed comparable prevalence (18–20%). Women in the middle wealth quintile had a numerically higher prevalence, though differences across wealth categories were not significant.

In bivariate and multivariable analyses (Table 2), low ferritin and elevated soluble transferrin receptor (sTfR) levels were strongly associated with increased odds of anemia. In contrast, overweight women had lower odds of anemia compared with women of normal BMI (AOR 0.31, *p* = 0.020). Women with four or more pregnancies also had lower odds of anemia (AOR 0.33, *p* = 0.009). Vitamin B12 deficiency was associated with lower odds of anemia (AOR 0.18, *p* = 0.050), an unexpected finding that should be interpreted cautiously. Maternal age, education, dietary diversity, antenatal care, iron–folic acid supplementation, parity, folate deficiency, and helminth infection were not significantly associated with anemia.

### 3.3. Characteristics of Preschool-Age Children (PSC, 6–59 Months)

A total of 500 preschool-age children (PSC, aged 6–59 months) were enrolled in the anemia study from various districts in the GBAO region. About one-fifth of these PSC belonged to the Shugnan district, while the remaining districts exhibited an equal distribution. Regarding gender distribution, there was a nearly even split between male (50.4%) and female (49.6%) children (Table 3).

A total sample of 390 preschool-age children (6–59 months) were assessed for anemia; district-specific data reveals nearly one-fifth (19%) of anemic children in Roshtkala, whereas the lowest proportion was reported by Rushan. Of the selected PSCs (6–59 months), nearly one-third (36.8%) were reported to be anemic, with their mothers belonging to the 20–24 age bracket. In addition, a higher proportion of anemia was seen in PSC (6–59 months) born to mothers with less education (18.1%) than those born to mothers with higher education (11.7%). Furthermore, 33.8% of children born to mothers with low maternal hemoglobin (<12 g/dL) were reported anemic; in addition, around one-fourth (25.4%) of children were born to mothers with low ferritin levels. When assessing anemia by gender, a substantial difference was observed: male children had a higher anemia rate (17.0%) than female children (13.9%). In addition, nearly one-fourth (26.5%) of children less than 2 years old were reported with anemia. 

### 3.4. Prevalence of Biochemical Indicators (PSC-6–59 Months)

Among PSC, 15.4% were anemic and 26.7% had low ferritin levels, indicating a substantial burden of iron deficiency. Iron deficiency anemia was present in 8.5% of children. Folic acid deficiency was highly prevalent, affecting 88.0% of the study population, while vitamin B12 deficiency was observed in 8.3%. Elevated transferrin receptor levels were detected in 59.8% of children, suggesting widespread iron-deficient erythropoiesis. Evidence of inflammation, reflected by elevated C-reactive protein, was found in 7.3% of children. Intestinal helminth infections were identified in 60.6% of stool samples, with pinworm detected in 33.5% and Ascaris in 27.2% of examined children.

### 3.5. Factors Associated with Anemia (PSC-6–59 Months)

In adjusted analyses (Table 4), low maternal education (AOR 2.35, *p* = 0.029) and maternal anemia (AOR 4.99, *p* = 0.001) were associated with increased odds of childhood anemia. Children younger than 24 months had more than twice the odds of anemia compared with older children (AOR 2.6, *p* = 0.016). Low ferritin was strongly associated with anemia (AOR 5.67, *p* < 0.001). Associations with maternal BMI, maternal ferritin, child sex, vitamin B12 deficiency, folate deficiency, stunting, wasting, and helminth infection were not statistically significant and should not be interpreted as causal.

## 4. Discussion

The prevalence of anemia among women of reproductive age (17.3%) and preschool children (15.4%) observed in this study was substantially lower than estimates reported in the Tajikistan National Nutrition Survey 2016 for the GBAO region [14]. Several factors may explain this difference. First, methodological differences exist between surveys, including hemoglobin measurement techniques, altitude adjustment procedures, and inflammation-adjusted ferritin estimation. Second, the current study was conducted five years later, during which nutrition programs, maternal health services, and food security initiatives may have improved. Third, selection bias related to blood sample availability cannot be excluded.

The 2016 National Nutrition Survey reported a high prevalence of inflammation, with elevated CRP observed in 82.5% of women and 64% of children. While hemoglobin was adjusted for altitude, iron biomarkers such as ferritin were adjusted for inflammation using CRP and AGP following standard WHO recommendations. High underlying inflammation may have increased the proportion of anemia inflammation (anemia of chronic disease), potentially contributing to the higher anemia prevalence observed in 2016. However, hemoglobin itself is not routinely adjusted for inflammation.

Inflammation has consistently been linked to higher rates of anemia beyond the impact of iron deficiency alone. For instance, a cross-sectional study of preschool children found that those with elevated C-reactive protein (CRP > 5 mg/L) had an anemia prevalence of 66%, compared to 40% in children with normal CRP. Similarly, children with elevated α-1-acid glycoprotein (AGP) showed higher anemia rates than those without, even after controlling for confounders [16]. Another study by Crooks et al. [17] concluded that a higher acute inflammatory response in illnesses like COVID-19 is associated with greater reductions in hemoglobin levels, illustrating anemia of acute inflammation and highlighting a pathway through which severe disease may worsen clinical outcomes. Broader survey data also indicate that inflammation can influence iron biomarkers, with ferritin levels varying with CRP and AGP, underscoring the need to consider these markers for accurate assessment of iron status in populations with high infection rates. These findings underscore the influence of inflammatory processes on hemoglobin and iron levels, likely through hepcidin-mediated iron sequestration and reduced erythropoiesis [18].

Iron deficiency emerged as the dominant determinant of anemia. Low ferritin and elevated soluble transferrin receptor levels were strongly associated with anemia in women, indicating depleted iron stores and functional iron deficiency. Similar associations were observed in preschool children, consistent with evidence from other low- and middle-income countries [19,20,21,22,23]. These findings reinforce iron deficiency as the primary biological driver of anemia in this population.

Maternal factors played a significant role in childhood anemia. Children born to mothers with lower educational attainment and those with maternal anemia had significantly higher odds of anemia, highlighting intergenerational and socioeconomic pathways. Our study corroborates with studies conducted in other developing countries [19,20,22,23]. Additionally, children younger than 24 months were particularly vulnerable, aligning with the critical first 1000 days window for growth and micronutrient sufficiency [21].

Unexpectedly, overweight status, higher gravidity, and vitamin B12 deficiency among women were associated with lower odds of anemia. These counterintuitive findings should be interpreted cautiously and may reflect residual confounding, dietary patterns associated with higher energy intake, or limitations of cross-sectional analysis. Importantly, obesity was not associated with anemia, suggesting a non-linear relationship between nutritional status and hemoglobin levels.

Helminth infections and inflammatory markers were not independently associated with anemia in this study, and, therefore, their role should not be overstated. While ferritin was adjusted for inflammation using CRP and AGP, inflammation itself was not modeled as an independent predictor, which limits the interpretation of inflammatory anemia pathways.

This study has several strengths. These include the use of comprehensive biochemical assessments with adjustment for altitude and inflammation, paired mother–child sampling that enabled examination of intergenerational determinants of anemia, and coverage of a geographically remote and historically understudied region of Tajikistan. The inclusion of multiple iron and inflammation biomarkers further strengthened the etiological assessment of anemia. However, the study also has limitations. Its cross-sectional design precludes causal inference, and biochemical data were unavailable for a subset of participants due to refusals or logistical constraints, potentially introducing selection bias. In addition, the sample size was not powered for precise district-level estimates, limiting the ability to detect small geographic differences in anemia prevalence [24].

## 5. Conclusions

Anemia prevalence among women of reproductive age and preschool children in GBAO, Tajikistan, was relatively low compared with previous national estimates but still poses a public health threat. Among women, anemia was primarily associated with iron deficiency markers, while overweight status, higher gravidity, and vitamin B12 deficiency were associated with lower odds of anemia. Among children, low ferritin, age under 24 months, maternal anemia, and lower maternal education were key determinants. These findings emphasize the importance of iron-focused interventions during early childhood, maternal nutrition, and educational empowerment, alongside cautious interpretation of unexpected associations observed in cross-sectional data.

## Figures and Tables

**Table 1:** Demographic, laboratory and household characteristics of WRA (15–49 years).

Women’s Background Characteristics	N (%)
	Overall
Total	500
** District:**	
Roshtkala	80 (16.0%)
Shugnan	100 (20.0%)
Ishkashim	80 (16.0%)
Murghob	80 (16.0%)
Rushan	80 (16.0%)
Khorog town	80 (16.0%)
** Age groups:**	
20–24	26 (5.2%)
25–29	107 (21.4%)
30–34	164 (32.8%)
35–39	124 (24.8%)
40–44	66 (13.2%)
45–49	13 (2.6%)
** Currently Married:**	
Yes	489 (97.8%)
No	11 (2.2%)
** Education:**	
None	1 (0.2%)
Incomplete secondary (grade 9)	24 (4.8%)
Secondary (grade 11)	165 (33.0%)
Technical college	98 (19.6%)
Incomplete higher education	10 (2.0%)
Higher education	201 (40.2%)
Others	1 (0.2%)
** Occupation:**	
University student	2 (0.4%)
Housewife	124 (24.8%)
Trader (shop, market)	5 (1.0%)
Business	3 (0.6%)
Self-employed in agriculture and non-agriculture sector	14 (2.8%)
Formal employee	197 (39.4%)
NGO employee	9 (1.8%)
Seasonal worker	2 (0.4%)
Unemployed	141 (28.2%)
Retired	1 (0.2%)
Others	2 (0.4%)
** Laboratory Indicators:**	
Anemia (Hb < 12 g/dL)	473 (17.3%)
Low Ferritin (<12 ng/mL)	359 (19.4%)
IDA (Hb < 12 g/dL & <12 ng/mL)	354 (11.6%)
Deficient Folic Acid (<3 ng/mL)	417 (61.6%)
Elevated Transferrin Receptor (>4.4 mg/L)	427 (22.0%)
Deficient Vitamin B12 (<191 pg./mL)	473 (11.2%)
Elevated CRP (>5)	417 (6.6%)
Stool Sample positive for Helminths Ascaris Pin Worm	475 (51.5%) 184 (38.7%) 65 (13.7%)
** Household Characteristics**	
** Drinking Water Sources**	
Improved sources	396 (79.2%)
Unimproved sources	104 (20.8%)
** Sanitation Facilities**	
Improved sanitation facility	459 (91.8%)
Unimproved sanitation facility	41 (8.1%)
** Food Insecurity Status**	
Food Secure	208 (41.6%)
Mildly food insecure	111 (22.2%)
Moderately food insecure	66 (13.2%)
Severely food insecure	115 (23.0%)

**Table 2:** Factors associated with anemia among non-pregnant women at age 15–49 years.

	N	Anemia (<12 g/dL)	Normal (≥12 g/dL)	Unadjusted OR (95% CI)	*p*-Value	Adjusted * OR (95% CI)	*p*-Value
Total	473	N = 82 (17.3%)	N = 391 (82.7%)				
**Household factors**							
**District**							
Roshtkala	75	15 (20%)	60 (80%)	Ref.			
Shugnan	94	19 (20%)	75 (80%)	1.013 (0.475–2.161)	0.973		
Ishkashim	78	9 (12%)	69 (88%)	0.522 (0.213–1.278)	0.155		
Murghob	67	12 (18%)	55 (82%)	0.873 (0.376–2.027)	0.752		
Rushan	79	12 (15%)	67 (85%)	0.716 (0.311–1.652)	0.434		
Khorog town	80	15 (19%)	65 (81%)	0.923 (0.416–2.048)	0.844		
**Wealth Index (quintiles)**							
Poorest	193	29 (15.0%)	164 (85.0%)	0.845 (0.488–1.463)	0.549		
Middle	95	21 (22.1%)	74 (77.9%)	1.357 (0.733–2.513)	0.332		
Richest	185	32 (17.3%)	153 (82.7%)	Ref.			
**Household food insecurity**							
Food Secure	198	32 (16.2%)	166 (83.8%)	Ref.			
Food Insecure	275	50 (18.2%)	225 (81.8%)	1.153 (0.708–1.876)	0.567		
**Maternal factors**							
**Age groups**							
20–24	26	6 (23.1%)	20 (76.9%)	Ref.		Ref.	
25–29	99	18 (18.2%)	81 (81.8%)	0.741 (0.26–2.107)	0.574	0.406 (0.092–1.788)	0.233
30–34	161	28 (17.4%)	133 (82.6%)	0.702 (0.258–1.906)	0.487	0.51 (0.126–2.07)	0.346
35–39	112	21 (18.8%)	91 (81.3%)	0.769 (0.275–2.151)	0.617	0.914 (0.211–3.959)	0.904
40–44	63	8 (12.7%)	55 (87.3%)	0.485 (0.15–1.571)	0.228	0.817 (0.156–4.287)	0.812
45–49	12	1 (8.3%)	11 (91.7%)	0.303 (0.032–2.85)	0.296	-	
**Maternal Education**							
Less than higher education	285	47 (16.5%)	238 (83.5%)	0.863 (0.533–1.398)	0.550		
Higher and above education	188	35 (18.6%)	153 (81.4%)	Ref.			
**Body Mass Index**							
Normal	291	58 (19.9%)	233 (80.1%)	Ref.		Ref.	
Underweight	42	7 (16.7%)	35 (83.3%)	0.803 (0.34–1.901)	0.618	1.426 (0.405–5.016)	0.581
Overweight	100	11 (11.0%)	89 (89.0%)	0.497 (0.249–0.989)	0.046	0.314 (0.118–0.833)	0.020
Obese	38	6 (15.8%)	32 (84.2%)	0.753 (0.301–1.887)	0.545	0.479 (0.111–2.069)	0.324
**Minimum Dietary Diversity for Women**							
MDDW ≥ 5	346	60 (17.3%)	286 (82.7%)	Ref.			
MDDW < 5	127	22 (17.3%)	105 (82.7%)	0.999 (0.584–1.709)	0.996		
**Iron folic acid supplementation**							
Yes	385	67 (17.4%)	318 (82.6%)	Ref.			
No	88	15 (17.0%)	73 (83.0%)	0.975 (0.527–1.804)	0.936		
**ANC visit**							
Yes	436	75 (17.2%)	361 (82.8%)	Ref.			
No	37	7 (18.9%)	30 (81.1%)	1.123 (0.476–2.653)	0.791		
**Number of pregnancies**							
<4	281	55 (19.6%)	226 (80.4%)	Ref.		Ref.	
≥4	192	27 (14.1%)	165 (85.9%)	0.672 (0.407–1.111)	0.122	0.326 (0.141–0.758)	0.009
**Number of deliveries**							
<4	376	66 (17.6%)	310 (82.4%)	Ref.			
≥4	97	16 (16.5%)	81 (83.5%)	0.928 (0.51–1.688)	0.806		
**Ferritin**							
Low Ferritin (<12 ng/mL)	79	41 (51.9%)	38 (48.1%)	13.05 (6.973–24.424)	<0.001	8.549 (3.791–19.276)	<0.001
Normal (≥12 ng/mL)	275	21 (7.6%)	254 (92.4%)	Ref.		Ref.	
**Folic acid**							
Deficient (<3 ng/mL)	241	41 (17.0%)	200 (83.0%)	1.058 (0.614–1.823)	0.840		
Not Deficient (≥3 pg/mL)	154	25 (16.2%)	129 (83.8%)	Ref.			
**Transferrin receptor levels**							
Normal (≤4.4)	317	30 (9.5%)	287 (90.5%)	Ref.			
Elevated (>4.4 mg/L)	87	40 (46.0%)	47 (54.0%)	8.142 (4.628–14.325)	<0.001	4.817 (2.107–11.014)	<0.001
**Vitamin B12**							
Deficient (<191 pg/mL)	47	4 (8.5%)	43 (91.5%)	0.409 (0.142–1.178)	0.098	0.18 (0.033–0.998)	0.050
Not Deficient (≥191 pg/mL)	367	68 (18.5%)	299 (81.5%)	Ref.		Ref.	
**Worm infestation**							
Worm infestation	66	9 (13.6%)	57 (86.4%)	0.772 (0.364–1.638)	0.501		
No Worm infestation	383	65 (17.0%)	318 (83.0%)	Ref.			

* Adjusted for maternal age, BMI, gravidity, ferritin, transferrin receptor, and vitamin B12.

**Table 3:** Background characteristics of PSC-6–59 months.

Total	N (%)
**District:**	
Roshtkala	80 (16.0%)
Shugnan	100 (20.0%)
Ishkashim	80 (16.0%)
Murghob	80 (16.0%)
Rushan	80 (16.0%)
Khorog town	80 (16.0%)
**Gender:**	
Male	252 (50.4%)
Female	248 (49.6%)
**Age groups:**	
6–11 months	41 (8.2%)
12–17 months	55 (11.0%)
18–23 months	64 (12.8%)
24–35 months	116 (23.2%)
36–47 months	122 (24.4%)
48–59 months	102 (20.4%)
**Laboratory Indicators:**	
Anemia (<11 g/dL)	390 (15.4%)
Low Ferritin (<12 ng/mL)	358 (26.7%)
IDA (Hb < 11 g/dL & <12 ng/mL)	329 (8.5%)
Deficient Folic Acid (<3 ng/mL)	351 (88.0%)
Elevated Transferrin Receptor (>4.4 mg/L)	361 (59.8%)
Deficient Vitamin B12 (<191 pg./mL)	362 (8.3%)
Elevated CRP (>5)	381 (7.3%)
Stool Sample positive for Helminths	478 (60.6%)
Ascaris Pin Worm	130 (27.2%) 160 (33.5%)

**Table 4:** Factors associated with anemia among preschool-age children (6–59 months).

Characteristics	N	Anemia (<11 gm/dL)	Normal (≥11 gm/dL)	Unadjusted OR (95% CI)	*p*-Value	Adjusted * OR (95% CI)	*p*-Value
	390	N = 60 (15.4%)	N = 330 (84.6%)				
**Household factors**							
**District**							
Roshtkala	72	14 (19%)	58 (81%)	Ref.			
Shugnan	66	9 (14%)	57 (86%)	0.654 (0.262–1.631)	0.363		
Ishkashim	60	10 (17%)	50 (83%)	0.829 (0.338–2.028)	0.681		
Murghob	35	6 (17%)	29 (83%)	0.857 (0.298–2.462)	0.775		
Rushan	79	10 (13%)	69 (87%)	0.6 (0.248–1.453)	0.258		
Khorog town	78	11 (14%)	67 (86%)	0.68 (0.287–1.614)	0.382		
**Wealth Index (quintiles)**							
Two Poorest	168	26 (15.5%)	142 (84.5%)	0.971 (0.527–1.789)	0.925		
Middle	77	11 (14.3%)	66 (85.7%)	0.884 (0.406–1.926)	0.756		
Two Richest	145	23 (15.9%)	122 (84.1%)	Ref.			
**Household food insecurity**							
Food Secure	159	25 (15.7%)	134 (84.3%)	Ref.			
Food Insecure	231	35 (15.2%)	196 (84.8%)	0.957 (0.548–1.673)	0.878		
**Maternal factors**							
**Maternal Age groups**							
20–24	19	7 (36.8%)	12 (63.2%)	Ref.		Ref.	
25–29	83	11 (13.3%)	72 (86.7%)	0.262 (0.085–0.809)	0.020	0.799 (0.16–3.99)	0.784
30–34	132	18 (13.6%)	114 (86.4%)	0.271 (0.094–0.778)	0.015	0.636 (0.139–2.898)	0.558
35–39	93	13 (14.0%)	80 (86.0%)	0.279 (0.093–0.838)	0.023	0.551 (0.117–2.589)	0.450
40–44	54	8 (14.8%)	46 (85.2%)	0.298 (0.09–0.987)	0.048	0.519 (0.094–2.846)	0.450
45–49	9	3 (33.3%)	6 (66.7%)	0.857 (0.161–4.554)	0.856	7.174 (0.525–98.092)	0.140
**Maternal Education**							
Less than higher education	227	41 (18.1%)	186 (81.9%)	1.671 (0.93–3.001)	0.086	2.35 (1.09–5.07)	0.029
Higher and above education	163	19 (11.7%)	144 (88.3%)	Ref.		Ref.	
**Maternal Hemoglobin**							
Anemia deficiency (<12 gm/dL)	65	22 (33.8%)	43 (66.2%)	3.797 (2.052–7.025)	<0.001	4.998 (2.019–12.373)	0.001
Normal (≥12 gm/dL)	320	38 (11.9%)	282 (88.1%)	Ref.		Ref.	
**Maternal Ferritin**							
Low Ferritin (<12 ng/mL)	67	17 (25.4%)	50 (74.6%)	1.599 (0.81–3.156)	0.176	0.655 (0.255–1.684)	0.380
Normal (≥12 ng/mL)	233	34 (14.6%)	199 (85.4%)	Ref.		Ref.	
**Body Mass Index**							
Normal	230	41 (17.8%)	189 (82.2%)	Ref.		Ref.	
Underweight	34	4 (11.8%)	30 (88.2%)	0.615 (0.205–1.84)	0.384	0.42 (0.091–1.93)	0.265
Overweight	91	10 (11.0%)	81 (89.0%)	0.569 (0.272–1.191)	0.135	0.554 (0.223–1.376)	0.203
Obese	33	5 (15.2%)	28 (84.8%)	0.823 (0.3–2.259)	0.706	0.516 (0.136–1.958)	0.331
**Child’s factors**							
**Gender**							
Male	188	32 (17.0%)	156 (83.0%)	Ref.			
Female	202	28 (13.9%)	174 (86.1%)	0.784 (0.452–1.361)	0.388		
**Age groups**							
<24 months	102	27 (26.5%)	75 (73.5%)	2.782 (1.573–4.919)	<0.001	2.6 (1.197–5.647)	0.016
≥24 months	288	33 (11.5%)	255 (88.5%)	Ref.		Ref.	
**Ferritin**							
Low Ferritin (<12 ng/mL)	77	24 (32.0%)	51 (68.0%)	5.429 (2.901–10.157)	<0.001	5.67 (2.647–12.145)	<0.001
Normal (≥12 ng/mL)	252	20 (9.7%)	187 (90.3%)	Ref.		Ref.	
**Vitamin B12**							
Deficient (<191 pg/mL	28	24 (85.7%)	4 (14.3%)	0.861 (0.286–2.594)	0.790		
Not Deficient (≥191 pg/mL)	296	248 (83.8%)	48 (16.2%)	Ref.			
**Folic acid**							
Deficient (<3 ng/mL)	272	230 (84.6%)	42 (15.4%)	0.913 (0.358–2.328)	0.849		
Not Deficient (≥3 pg/mL)	36	30 (83.3%)	6 (16.7%)	Ref.			
**Stunting (Height-for-Age)**							
Normal	311	50 (16.1%)	261 (83.9%)	Ref.			
Stunting	60	8 (13.3%)	52 (86.7%)	0.803 (0.36–1.794)	0.593		
**Underweight (Weight-for-age)**							
Normal	345	55 (15.9%)	290 (84.1%)	Ref.			
Underweight	33	4 (12.1%)	29 (87.9%)	0.727 (0.246–2.151)	0.565		
**Wasting (Weight-for-length/height)**							
Normal	365	58 (15.9%)	307 (84.1%)	Ref.			
Wasting	13	1 (7.7%)	12 (92.3%)	0.441 (0.056–3.458)	0.436		
**Worm infestation**							
Worm infestation	133	22 (16.5%)	111 (83.5%)	1.213 (0.676–2.174)	0.518		
No Worm infestation	242	34 (14.0%)	208 (86.0%)	Ref.			
**Ever given Iron Syrup in the last six months**							
Yes	260	42 (16.2%)	218 (83.8%)	Ref.			
No	130	18 (13.8%)	112 (86.2%)	0.834 (0.459–1.516)	0.552		

* Adjusted for maternal education, maternal hemoglobin status, child age (<24 months), and ferritin.

## Data Availability

The data presented in this study will be made by the corresponding authors on request.
